# A New Natural Ceramide from *Trollius chinensis* Bunge

**DOI:** 10.3390/molecules15107467

**Published:** 2010-10-25

**Authors:** Ru-Feng Wang, Rui-Ning Liu, Tong Zhang, Tao Wu

**Affiliations:** 1 School of Chinese Medicine, Beijing University of Chinese Medicine, Beijing 100102, China; E-Mail: wangrufeng@tsinghua.org.cn (R-F.W.); 2 Department of Pharmacy, First Hospital of Tsinghua University, Beijing 100016, China; E-Mail: lrnthu@yahoo.com.cn (R-N.L.); 3 Experiment Center for Teaching and Learning, Shanghai University of Traditional Chinese Medicine, Shanghai 201203, China

**Keywords:** *Trollius chinensis*, constituents, ceramide

## Abstract

A new natural product named trolliamide was isolated from *Trollius chinensis* Bunge. Its structure was determined as 2-hydroxy-tetracosanoic acid(2,3-dihydroxy-1-hydroxymethyl-heptadec-7-enyl)-amide by spectroscopic methods, including UV, IR, MS and NMR. This is the first report of a ceramide isolated from *Trollius chinensis*.

## Introduction

*Trollius chinensis* Bunge (Ranunculaceae) is a perennial herb widely distributed in northern China [1]. Its flowers, known as Jinlianhua in China, have been used by Chinese people for a long time to treat respiratory infections, pharyngitis, tonsillitis, and bronchitis [2]. Phytochemical studies reported that these flowers mainly contain flavonoids such as vitexin, orientin, isoswertiajaponin and isoswertisin, phenolic acids such as veratric acid and proglobeflowery acid, as well as alkaloids such as trollisine, and some literature has demonstrated flavonoids and phenolic acids as their effective constituents [3,4,5]. This paper deals with the isolation and structural elucidation of a new natural ceramide product named trolliamide, which was isolated as a chemical entity from this species for the first time.

## Results and Discussion

The compound was obtained as a white powder with the melting point of 116–118 °C. Its molecular formula was determined as C_42_H_83_NO_5_ by negative HRESIMS at *m/z* 680.6279 [M-H]^-^. On thin layer chromatography it was detected as a weak dark spot at 254 nm and a brown spot after spraying with 10% H_2_SO_4_. Its UV spectrum exhibited a strong peak at 243 nm, which is a characteristic band of amides. The IR spectrum demonstrated the existence of hydroxyl (3,215 cm^−1^), secondary amino (3,334 cm^−1^) and amide groups (1,622 cm^−1^) in addition to aliphatic chains (2,919 and 1,850 cm^−1^). All this evidence suggested that this compound is a ceramide, which was confirmed by its ^1^H- and ^13^C- NMR spectra. In the ^13^C NMR spectrum (Table 1), the signals of three characteristic ceramide groups appeared at *δ* 175.2 (carbonyl), 52.9 (the carbon connecting the nitrogen atom), and 29.9 (the (CH_2_)_n_ signals). In the ^1^H-NMR spectrum (Table 1), the strong (CH_2_)_n_ signals at *δ* 1.30 and two terminal methyl signals at *δ* 0.87 also confirmed the ceramidic nature of this compound. 

**Table 1:** ^1^H- and ^13^C-NMR spectral data (in C_5_D_5_N) of trolliamide.

No.	^1^H ^a)^*δ*, *J* in Hz	^13^C ^b)^ *δ*
1	4.48(1H, dd, 10.7, 6.7 )4.43(1H, dd, 10.7, 4.5)	62.0
2	5.11(1H, m)	52.9
3	4.61(1H, m)	76.8
4	4.31(1H, m)	72.9
5	2.00(2H, m)	33.8
6	1.74(2H, m)	26.7
7	2.16(2H, m)	33.3
8	5.53(1H, m)	130.8
9	5.53(1H, m)	130.7
10	1.99(2H, m)	33.0
11-15	1.30 -1.24	30.3-29.5
16	1.30 -1.24	32.1
17	1.30 -1.24	22.9
18	0.87(3H, t, 6.0)	14.3
1ʹ	-	175.2
2ʹ	4.35(1H, m)	72.4
3ʹ	1.98(2H, m)	35.7
4ʹ	1.76(2H, m)	25.8
5ʹ-21ʹ	1.30-1.24	30.3-29.5
22ʹ	1.30-1.24	32.1
23ʹ	1.30-1.24	22.9
24ʹ	0.87(3H, t, 6.0)	14.3
NH	8.59(1H, d, 9.0)	

^a)^ 300 MHz; ^b)^ 75 MHz

**Figure 1:**
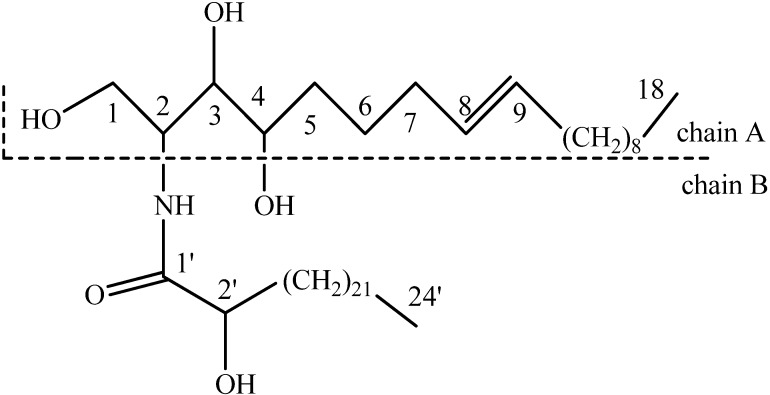
The structure of trolliamide.

The entire structure of this compound (Figure 1) was established by 1D and 2D NMR spectra in combination with the MS data. With the help of the HMQC spectrum, the ^1^H-NMR signal at *δ* 5.11(1H, m) was assignable to H-2, which correlated with signals at *δ* 4.48 (1H, dd, *J* = 10.7 Hz, 6.7 Hz, H-1), 4.43(1H, dd, *J* = 10.7 Hz, 4.5 Hz, H-1) and 4.61(1H, m, H-3) in the ^1^H-^1^H COSY spectrum. C-1 and C-3 were assigned to the signals at *δ* 62.0 and 76.8, respectively, on the basis of the HMQC spectrum. C-3 correlated with the signal at *δ* 4.31 (1H, m, H-4) in the HMBC spectrum, indicating C-3 and C-4 of chain A were hydroxylated. The C-2ʹ (*δ* 72.4) of chain B was also demonstrated to be hydroxylated from the correlation between the carbonyl signal at *δ* 175.2(C-1ʹ) and *δ* 4.35 (1H, m, H-2ʹ). Thus, the structural fragments were confirmed based on above information. The full composition of chains A and B was determined on the basis of fragmentation pattern in the EI-MS spectrum. In the EI-MS spectrum, the molecular ion peak at *m/z* 681 and a dehydrated molecular ion peak at *m/z* 663 also confirmed its molecular weight. In accordance with the literature [6], the length of chains A and B was determined based on *m/z* 280 (the fragment ion of chain A after dehydration) and *m/z* 384 (the fragment ion of chain B after McLafferty rearrangement of its carbonyl). The ^13^C-NMR signals at *δ* 130.8 (C-8) and 130.7 (C-9) demonstrated the presence of a double bond in the molecule. This double bond was determined to be *trans* on the grounds of the allylic chemical shifts at *δ* 33.3(C-7) and 33.0 (C-10) [6], and was located at C-8 and C-9 of chain A because of the HMBC correlation between H-6 [*δ* 1.74 (2H, m)] and C-8, as well as between H-4 and C-6 (*δ* 26.7). In summary, the structure was identified as 2-hydroxy-tetracosanoic acid (2,3-dihydroxy-1-hydroxymethyl-heptadec-7-enyl)-amide. Although this compound was first discovered by Kraus when he analyzed the mixture of the extract of *Urtica dioica* through GC-MS [7], as far as we know, it is the first time that this compound is isolated as the pure entity from plants.

## Experimental

### General

The melting point was measured on an XT-4A digital melting point apparatus without correction. The IR spectrum (film) was recorded on a Thermo Nicolet Nexus 470 FT-IR spectrophotometer. The UV spectrum was obtained on a Varian Cary Eclipse 300 spectrophotometer using chloroform as the solvent. NMR spectra were determined on a JEOL JNM 300 spectrometer (at 300 MHz for ^1^H-NMR and 75 MHz for ^13^C-NMR) with the solvent as internal standard. HR-ESI-MS spectrum was measured on a Bruker APEX mass spectrometer. Silica gel (200–300 mesh) used in column chromatography was provided by Qingdao Marine Chemistry Co. Ltd. Analytical TLC was conducted on silica gel GF_254_ plates (manufactured by Merck) and preparative TLC was performed on self-made silica gel GF_60_ plates.

### Plant Material

The flowers of *Trollius chinensis* were obtained from the Anguo Chinese crude drug market in Hebei Province of China, and authenticated by Dr. Rufeng Wang as flowers of the title plant. A voucher specimen was deposited in the herbarium of School of Chinese Medicine, Beijing University of Chinese Medicine.

### Extraction and Isolation

The dried flowers of *T. chinensis* (2 kg) were extracted with 95% aqueous ethanol (20 L) under reflux, and the resulting solution was concentrated *in vacuo* to yield 331 g of crude extract. The crude extract was suspended in water (1 L), and petroleum ether, ethyl acetate and *n*-butanol (each 1 L × 3 times) were added successively to partition it into four parts, namely, petroleum ether (92 g), ethyl acetate (48 g), *n*-butanol (82 g) and water soluble (109 g) parts. The petroleum ether soluble part was subjected to silica gel column chromatography eluted with a petroleum ether-acetone gradient from 20:1 to 1:1 to afford fractions A to F, of which fraction C was further separated by silica gel column chromatography eluted with petroleum ether-acetone from 15:1 to 1:1 to yield subfractions C-1–C-9. Subfraction C-4 was separated by preparative TLC developed by petroleum ether-acetone 3:1 to yield the title compound (25 mg). 

### Trolliamide

White powder, mp116–118 °C; negative HRESIMS *m/z* 680.6279 [M+H]^-^ (calcd for C_42_H_82_NO_5_, 680.6271); EI-MS *m/z*: 681 (M)^+^, 663 (M-H_2_O)^+^, 456, 439, 408, 384, 356, 280, 262, 239, 185, 155, 139, 129, 125, 98, 97, 83, 60, 57; UV (CDCl_3_) λ_max_: 243 nm; IR (KBr) ν_max_: 3,334 (NH), 3,215 (OH), 2,919, 2,850 (CH), 1,622 (C=O), 1,545, 1,467 (-CH=CH-), 1.357, 1.271, 1.067, 1.021, 964, 723 cm^−1^;^ 1^H- and ^13^C-NMR(C_5_D_5_N), see Table 1.

## Conclusions

In summary, this compound is identified as the structure described above by its spectral data including UV, IR, MS, and 1D and 2D NMR. This is the first time that a ceramide is isolated and identified from *Trollius chinensis* Bunge, which may be in favor of the advanced research and development of this traditional herbal drug. 
